# Domestic violence during the COVID-19 confinement: do victims feel more socially isolated?

**DOI:** 10.1186/s13690-021-00765-3

**Published:** 2022-01-25

**Authors:** Sabine Drieskens, Elise Braekman, Karin De Ridder, Lydia Gisle, Rana Charafeddine, Lize Hermans, Stefaan Demarest

**Affiliations:** Scientific Direction Epidemiology and public health, Sciensano, J. Wytsmanstreet 14, 1050, Brussels, Belgium

**Keywords:** Domestic violence, Social isolation, COVID-19, Confinement, Health survey, Social support, Loneliness

## Abstract

**Background:**

Since March 13th 2020, confinement measures have been introduced in Belgium to curb the spread of the coronavirus (COVID-19). These measures also have an impact on people’s daily life (closure of school/businesses, teleworking, recommendation to stay at home). This can cause stress on social, economic and psychological levels and thereby can trigger domestic violence. Besides, confinement also fosters social isolation, which can complicate help seeking behaviour. The aim of this study is to determine the prevalence of domestic violence during the coronavirus crisis and to assess whether there is an association between domestic violence and social isolation.

**Methods:**

Several online COVID-19 Health Surveys were organised among Belgian residents aged 18+ via snowball sampling. This study is based on the second (April 2020) and the sixth survey (March 2021). After excluding 1-person households and missing data, the sample size was respectively 25,251 and 12,589. Weighted prevalence of domestic violence was evaluated for the two surveys. The association (OR; 95% CI; *p*-value) between domestic violence and subjective social isolation was assessed with logistic regression stratified by survey and adjusted for covariates.

**Results:**

In April 2020, 4.0% of the adult population reported being a victim of domestic violence (1.2% in the Health Interview Survey 2018); in March 2021, this was 6.2%. In April 2020, victims of domestic violence had higher odds of being unsatisfied with their social contacts (OR = 1.25; 95% CI: 1.08–1.44; *p* < .05), weak social support (OR = 2.26; 95% CI: 1.97–2.58; *p* < .0001) and having less confidence in health care services (OR = 1.38; 95% CI: 1.13–1.71; *p* < .05). In March 2021, victims had higher odds of being unsatisfied with their social contacts (OR = 1.30; 95% CI: 1.08–1.56; *p* < .05) and weak social support (OR = 2.41; 95% CI: 2.04–2.84; *p* < .0001), and social (OR = 2.64; 95% CI: 2.23–3.13; *p* < .0001) and emotional loneliness (OR = 2.22; 95% CI: 1.80–2.73; p < .0001).

**Conclusions:**

More people have reported domestic violence since the start of the coronavirus crisis than did in 2018. An association between domestic violence and social isolation was determined. Although confinement is needed to counteract the virus, it can put people in a dangerous situation since they do not get the help they need. Therefore, adequate support is essential.

## Background

Domestic violence is generally defined as violence between intimate partners, but also as violence against household members (children, parents and elderly) [1–3]. Although women are sometimes the perpetrator, they are most often the victim [4]. Domestic violence is not only a breach of human rights, it is also a major public health problem [4–7]. Since it occurs in different forms (physical, psychological and sexual violence), its health impact varies from mental problems (including depression, anxiety and suicide attempts) to physical injuries [7, 8], which in turn may lead to a more frequent use of health care services [9]. Domestic violence can also be fatal [8]. Though psychological violence is more prevalent, it more often remains undetected [2].

Prevalence rates of domestic violence on a national level are rather scarce and complicated to gather. Moreover, discrepancies in the definition (form of violence and target group) and in the methodology applied in studies make comparisons difficult [2]. Commonly used sources to obtain figures on domestic violence are clinical studies, police and hospital records, but those figures cannot be generalized to the overall population. A scientific review of mostly U.S. surveys on violence has shown that the past year prevalence of intimate partner violence against women varies between 2 and 12% [10]. According to the 2018 national Health Interview Survey (HIS) of Belgium, 1% of the population, aged 18 years and older reported being a victim of domestic violence in the 12 months preceding the survey [11]. It has been shown that a HIS is an appropriate tool to deliver population-based prevalence of victimization and to study the association between domestic violence and different health outcomes including physical, mental and social dimensions [2].

Since the start of the COVID-19 pandemic in 2020, confinement measures have been taken globally to curb the spread of the coronavirus and to relieve the health care system [1, 12–14]. These measures had a tremendous impact on people’s daily life, such as closure of schools and businesses, teleworking and the recommendation to stay at home [1, 13–15]. This can cause stress on social, economic and psychological levels and thereby can trigger violence [16]. Accordingly, domestic violence has extensively been reported since the beginning of the coronavirus crisis: i.e. an increase of 30% in France, 25% in Argentina, 30% in Cyprus, 33% in Singapore [1, 12, 13, 17]. This may also be the case in Belgium as the number of calls regarding domestic violence to the helplines increased by 70% since the beginning of this crisis [18].

Confinement measures foster social isolation [15, 16] as people have to restrict social contacts with external family members, friends, neighbors and colleagues, and have limited access to social and health care services [7, 12, 13, 15–17, 19]. Social isolation can be detrimental especially for victims of domestic violence since seeking help becomes more difficult [7, 12, 14, 17, 19, 20]. Moreover, it is harder for the victims to report domestic violence because they are trapped with the perpetrator [12, 15, 18]. Besides, perpetrators commonly use social isolation as a tactic to deprive their victims of their support network. In this sense, the confinement measures only play to the advantage of the perpetrators [13, 14, 17, 21].

In this study, we aim to examine the evolution of domestic violence throughout the coronavirus crisis and to assess the association between confinement and domestic violence by using data from an online survey organised among the adult population living in Belgium conducted 6 weeks and 1 year after the introduction of the confinement measures. We hypothesize that the prevalence of domestic violence is higher during the confinement period compared to the prevalence measured in the HIS 2018. Additionally, we hypothesize that during the confinement, victims of domestic violence have experienced more social isolations than non-victims.

## Methods

### Survey methodology

Since the introduction of the confinement measures in Belgium by the National Security Council on the 13th of March 2020 [22], the Belgian Institute of Public health Sciensano organised a series of online COVID-19 Health Surveys to evaluate the impact of the coronavirus crisis on people’s daily life. Even though the restriction measures were gradually loosened starting from 4 May 2020, the rise of SARS-Cov-2 infections in the second and third COVID-19 waves meant that the measures had to be tightened up again on 18 October 2020 [23] and 26 March 2021 [24] respectively. However, the measures that were in place during the first COVID-19 wave were the most restrictive. The questions on domestic violence were asked in the second survey (launched from 16 to 22 April 2020 – during the first COVID-19 wave) and in the sixth survey (launched from 17 to 25 March 2021 – just before the third COVID-19 wave). All the surveys were developed using LimeSurvey version 3. The average completion time was 15 min.

The link to the survey was, among others, announced on Sciensano’s and other organization’s websites, as well as through the press and social media. Moreover, an invitation email was sent to contacts of Sciensano employees and to the participants who indicated in a given survey that they agreed to be invited for the next one. Furthermore, they were asked to spread the link of the survey to family, friends and acquaintances, the so called snowball sampling [25]. Each cross-sectional survey received the approval of the Ethics Committee of the University of Ghent (BC-07544), in accordance with the General Data Protection Regulation (GDPR) and the Declaration of Helsinki [26, 27]. Only Belgian residents aged 18 years or older who gave their consent were allowed to take part in the survey [25].

### Study population

**Fig. 1:**
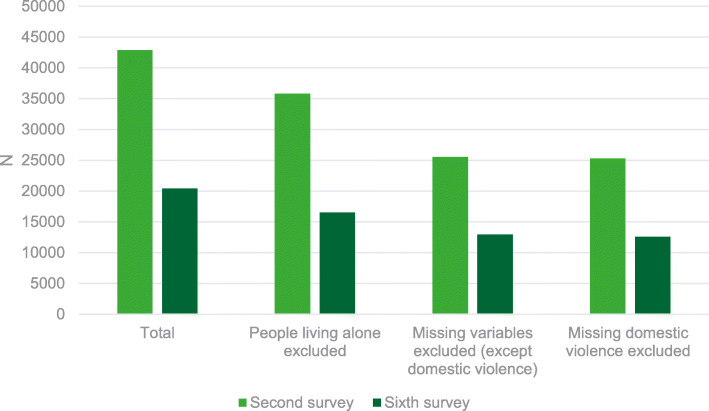
From total sample to study sample, second and sixth COVID-19 Health Survey, Belgium 2020- 2021

The total number of participants in the second COVID-19 Health Survey (April 2020) was 42,895. As those living alone were not asked questions on domestic violence, these participants were excluded (N sample = 35,800). Also participants with missing data on the sociodemographic and health covariates and social isolation indicators (N sample = 25,532), as well as participants with missing data on domestic violence were excluded. The final study sample consisted of 25,251 individuals aged 18 years and older. In the sixth survey (March 2021) these numbers were respectively 20,410 (total participants), 16,520 (after excluding people living alone), 12,909 (after excluding missing sociodemographic and health covariates and social isolation indicators), 12,589 (after excluding missing domestic violence variable) (Fig. 1). People may have participated in both surveys, but since it is not a follow-up survey, the number of those people is not known. Post-stratification weights, based on the composition of the population from the “Labour Force Survey 2018”, were applied to the COVID-19 Health Survey samples to correct for the distribution in terms of region, gender, age group and educational attainment [25].

### Variables

Tables 1, 2 and 3 give an overview of the questions used in both surveys and the corresponding variables (covariates and indicators). Both the independent as well as the dependent indicators were dichotomised.

**Table 1:** Overview of the questions and the corresponding covariates of the second and the sixth COVID-19 Health Survey, Belgium 2020–2021

Questions second survey	Questions sixth survey	Sociodemographic and health covariates
What is your year of birth?	Idem	Age group 1. 18–34 years 2. 35–54 years 3. 55+ years
What is you gender? 1. Man 2. Women 3. Other	Idem	Gender 1. Men 2. Women - (excluded)
What type of household do you live in? 1. Living alone 2. As couple without child (ren) 3. As a couple, with child (ren) 4. Living alone with child (ren) 5. With my parent(s), family, friends or acquaintances 6. Other	Idem	Household composition - (excluded) 2. Couple, without child (ren) 3. Couple, with child (ren) 4. Living alone with child (ren) 5. Living with parents, family, … 6. Other
Including yourself, how many people live in your household?	How many people live in your household, including yourself?	Household size 1. 2–4 persons 2. 5 persons or more
What is the highest level of education degree you have completed? 1. No degree 2. Primary education 3. Secondary education 4. Bachelor 5. Master 6. Doctorate: PhD 7. Other	Idem	Educational attainment 1. Low (answer categories 1–3) 2. High (answer categories 4–6) - (answer category 7 excluded)
To what extent does the coronavirus (COVID-19) epidemic have an impact on your household’s monthly net income 1. No impact 2. Limited impact (limited loss of income) 3. Significant impact (significant loss of income) 4. Serious impact (serious loss of income)	On the following scale, to what extent does the corona crisis still impact the following domains of your life today, considering that − 5 = most negative impact, 0 = no impact and + 5 = the most positive impact? 11 domains including income	Impact of COVID-19 on income 1. No (or positive) impact - Survey 2: answer category 1 - Survey 6: from + 5 to 0 2. Negative impact (loss) - Survey 2: answer categories 2–4 - Survey 6: − 1 to − 5
Health related quality of life: standardized instrument developed by the European EuroQol [34] concerning 5 dimensions: problems of mobility, self-care, performance of the usual activities, pain/discomfort and anxiety/depression	Idem	Reporting no health problems (EQ-5D) 1. Yes (report a problem in at least one of the dimensions) 2. No (no problem on all dimensions)

**Table 2:** Overview of the questions and the corresponding indicators of the second and the sixth COVID-19 Health Survey, Belgium 2020–2021

Questions second survey	Questions sixth survey	Independent indicators
Since 13 March 2020, have you experienced verbal/psychological violence (e.g. insults, ridicule, humiliation), physical violence (e.g. being beaten, shaken up) or sexual violence (e.g. forced sexual acts, rape) within in your family? 1. No, and never before 2. No, but before that 3. Yes, but less than usual 4. Yes, same as usual 5. Yes, and more than usual	In the past 12 months, have you experienced verbal/psychological violence (e.g. insults, ridicule, humiliation), physical violence (e.g. being beaten, shaken up) or sexual violence (e.g. forced sexual acts, rape) within in your family? 1. No, and never before 2. No, but before that 3. Yes, but less than usual 4. Yes, same as usual 5. Yes, and more than usual	Victim of domestic violence 1. Yes (answer categories 3–5) 2. No (answer categories 1–2)
If yes	If yes	
Since 13 March 2020, what type of violence have you experienced within your family? - Verbal/psychological violence: 1. Yes – 2. No - Physical violence: 1. Yes – 2. No - Sexual violence: 1. Yes – 2. No	In the past 12 months, what type of violence have you experienced within your family? - Verbal/psychological violence: 1. Yes – 2. No - Physical violence: 1. Yes – 2. No - Sexual violence: 1. Yes – 2. No	Victim of psychological violence within the family 1. Yes 2. No Victim of physical violence (including sexual violence) within the family 1. Yes 2. No

**Table 3:** Overview of the questions and the corresponding indicators of the second and the sixth COVID-19 Health Survey, Belgium 2020–2021

Questions second survey	Questions sixth survey	Subjective social isolation dependent indicators
Based on what you have seen, read or heard, how confident are you that the following institutions are dealing well with the coronavirus (COVID-19) epidemic? “The health care services (doctors, hospitals, …)” 1. Very confident 2. Less confident 3. No opinion	Not included	Confidence in health care services 1. Very confident 2. Less confident - (answer category 3 excluded)
How do you judge your social contacts of the last 2 weeks? 1. Really satisfied 2. Rather satisfied 3. Rather unsatisfied 4. Really unsatisfied	Idem	Satisfaction with social contacts 1. (Rather) unsatisfied (answer cat. 3–4) 2. (Rather) satisfied (answer categories 1–2)
Over the last 2 weeks, how many people were so close to you that you could count on them if you would have had serious personal problems? 1. None 2. 1 or 2 3. 3–5 4. 6 or more	Idem	
Over the last 2 weeks, how much concern have people shown in what you were doing? 1. A lot of concern and interest 2. Some concern and interest 3. Uncertain 4. Little concern and interest 5. No concern and interest	Idem	Quality of social support derived from OSLO social support scale (OSSS-3) [35] 1. Weak support 2. Moderate/strong support
Over the last 2 weeks, how easy was it to get practical help from neighbours if you should have needed it? 1. Very easy 2. Easy 3. Possible 4. Difficult 5. Very difficult	Idem	
Not included	Below you find some statements about you and the people around you. Please score how valid the statement is for you. 01. I experience a general sense of emptiness 02. There are plenty of people I can rely on when I have problems 03. There are many people I can trust completely 04. There are enough people I feel close to 05. I miss having people around me 06. I often feel rejected With the answer categories: 1. Yes! – 2. Yes – 3. More or less – 4. No – 5. No!	Social loneliness derived from the De Jong-Gierveld scale [36] (based on the items 02, 03 and 04) 0. Not lonely 1. Lonely Emotional loneliness derived from the De Jong-Gierveld scale [36] (based on the items 01, 05 and 06) 0. Not lonely 1. Lonely

To assess whether during the confinement victims of domestic violence experienced more social isolation than nonvictims, we used a series of social isolation indicators as the dependent indicators. The definition of social isolation can vary from a simple description, like ‘the absence of contacts’, to a more complex one which comprises multiple dimensions concerning ‘feelings related to (the absence of) contacts, such as social support, belongingness, fulfillment and engagement’ [28]. An intermediate definition of social isolation which is bi-dimensional, makes a distinction between an objective and a subjective component ﻿[28–31]. Objective social isolation (or social disconnectedness) is defined as having a small social network (e.g. few close friends and relatives) [28–31] and having limited involvement in social activities [29, 30]. Conversely, subjective social isolation (or perceived isolation) is defined by a perceived lack of social support and a feeling of loneliness [28–30]. Gierveld and Tilburg [31] describe loneliness as “the expression of a negative feeling of missing relationships with others”. All five social isolation indicators used in this study concern subjective social isolation and refer to the survey questions evaluating the respondent’s satisfaction with social contacts, perceived quality of social support, trust in health care services, and social and emotional loneliness. The subjective social indicator ‘confidence in health care services’ may seems as an outsider, but those services are important for victims of domestic violence in the search for support and so having trust in them plays a crucial role here.

Being a victim of domestic violence on either April 2020 or March 2021 (that is 6 weeks or 1 year after the introduction of the confinement measures) was used as the independant variable to measure domestic violence. A number of sociodemographic and health variables have been accounted for as covariates as they are related to both domestic violence and social isolation [2, 8, 12, 13, 15–17, 19, 32, 36]. These covariates are: gender, age group, educational attainment, household composition and size, income loss and quality of life.

### Data analysis

First, the weighted prevalence of domestic violence and type of violence was assessed for the two time periods (i.e. 6 weeks and 1 year after the introduction of the confinement measures). We assessed the difference with the situation prior to the coronavirus crisis by comparing the odds of reporting being a victim in the HIS 2018 with the odds of being a victim in the COVID-19 Health Surveys while controlling for gender, age group and educational attainment.

Since the prevalence of domestic violence and its association with social isolation may differ by survey, the following analyses were stratified by survey and conducted on the final study samples of each of the two COVID-19 Health Surveys. The weighted frequency of the covariates and the subjective social isolation indicators were determined for the victims and the nonvictims of domestic violence. The association between domestic violence and subjective social isolation was assessed based on the adjusted ORs (95% CIs and *P*-values). The adjusted OR of each subjective social isolation indicator was controlled for all the covariates. Five different logistic regressions were modelled. The goodness-of-fit of these models was measured by the Hosmer and Leweshow test, whereby a large *p*-value (closer to 1) indicates a good fit and a *p* < .05 a poor fit to the data [37].

All the analysis were performed with SAS® 9.4. For the frequency analysis the post-stratification weights were taken into account (PROC SURVEYFREQ). Since the Hosmer and Leweshow test is inappropriate when applying the sample design, logistic regression analysis were conducted (PROC LOGISTIC) to model the association between domestic violence and social isolation.

## Results

After 6 weeks of confinement, 4.0% of the population aged 18 years and older and not living alone had indicated having been a victim of domestic violence (compared to 1.2% in the HIS 2018). The prevalence increased to 6.2% 1 year after the coronavirus crisis, however keep in mind that it concerns two different time periods (6 weeks versus 1 year). These figures are significantly higher than the prevalence shown in the HIS 2018 (HIS 2018: OR = reference - second COVID-19 Health Survey: OR = 3.06; 95% CI: 2.10–4.48; *p* < .0001 - sixth COVID-19 Health Survey: OR = 5.00; 95% CI: 3.33–7.36; p < .0001). In most cases, victims reported psychological violence (second COVID-19 Health Survey: 93.6%; sixth COVID-19 Health Survey: 95.6%), but in almost 1 in 5 cases (second COVID-19 Health Survey: 16.6%; sixth COVID-19 Health Survey: 19.1%) physical violence (including sexual violence) was also involved.

Table 4 gives an overview of the characteristics of the victims and the nonvictims of domestic violence by survey. Participants reporting being a victim were more prevalent among young adults aged 18–34 years, individuals living with their parents and family, larger households (5 members or more), individuals experiencing a negative impact of COVID-19 on their incomes and individuals having at least one health problem. Victims more often reported weak social support, having less confidence in health care services and feeling socially lonely.

**Table 4:** Distribution (weighted prevalence) of the sociodemographic and health covariates and the subjective social isolation indicators by victims and nonvictims of domestic violence, stratified by the second and sixth COVID-19 Health Survey, Belgium 2020–2021

	Second survey (April 2020)		Sixth survey (March 2021)	
	Victims (%) (*N* = 933)	Nonvictims (%) (*N* = 24,318)	Victims (%) (*N* = 636)	Nonvictims (%) (*N* = 11,953)
Age group				
18–34 years	41.5	28.3	49.2	28.6
35–54 years	37.4	35.3	31.8	34.9
55+ years	21.1	36.4	19.0	36.5
Gender				
Men	47.2	50.4	41.0	49.3
Women	52.8	49.6	59.0	50.7
Household composition				
Couple, without child (ren)	19.9	41.2	23.0	42.3
Couple, with child (ren)	39.5	35.4	31.1	34.0
Living alone with child (ren)	5.2	5.5	5.8	5.0
Living with parents, family, …	33.2	16.8	39.2	17.7
Other	2.2	1.1	0.9	1.0
Household size				
2–4 persons	80.0	89.8	84.9	89.9
5 persons or more	20.0	10.2	15.1	10.1
Educational attainment				
Low	65.7	62.3	69.5	63.9
High	34.3	37.7	30.5	36.1
Impact of COVID-19 on income				
No (or positive) impact	42.3	59.1	74.2	82.7
Negative impact (loss)	57.7	40.9	25.8	17.3
Health problem				
No health problem	10.8	28.3	12.5	29.2
At least one health problem	89.2	71.7	87.5	70.8
Satisfied with social contact				
(Rather) unsatisfied	69.9	62.9	76.4	63.2
(Rather) satisfied	30.1	37.1	23.6	36.8
Quality of social support				
Weak support	50.6	28.5	53.0	32.9
Moderate/strong support	49.4	71.5	47.0	67.1
Confidence in health care services				
Very confident	86.0	92.6	–	–
Less confident	14.0	7.4	–	–
Social loneliness				
Not lonely	–	–	34.0	60.6
Lonely	–	–	66.0	39.4
Emotional loneliness				
Not lonely	–	–	14.0	34.6
Lonely	–	–	86.0	65.4

Overall, all the models had a good fit (Table 5). Table 6 shows that 6 weeks after the introduction of the confinement measures, victims of domestic violence had significantly higher odds of being unsatisfied with their social contacts (OR = 1.25; 95% CI: 1.08–1.44); *p* < .05) and a perceived weak social support (OR = 2.26; 95% CI: 1.97–2.58; *p* < .0001) than nonvictims. One year after the introduction of the confinement measures, these odds were still significantly higher (respectively OR = 1.30; 95% CI: 1.08–1.56; *p* < .05 and OR = 2.41; 95% CI: 2.04–2.84; p < .0001) than for nonvictims. In the first 6 weeks of confinement, victims of domestic violence also had significantly higher odds of lacking confidence in health care services (OR = 1.38; 95% CI: 1.13–1.71; p < .05) than nonvictims. The indicator for having less confidence in health care services was only available in April 2020; while the indicators for feelings of loneliness were only available in March 2021. One year after the introduction of the confinement measures, victims of domestic violence had significantly higher odds of perceived social (OR = 2.64; 95% CI: 2.23–3.13; *p* < .0001) and emotional loneliness (OR = 2.22; 95% CI: 1.80–2.73; p < .0001) than nonvictims.

**Table 5:** Goodness-of-fit of the different models (by survey and subjective social isolation indicator) with the Hosmer and Lemeshow test

Social isolation indicators by survey	Χ^**2**^	DoF	***p***-value
*Second COVID-19 Health Survey*			
Satisfied with social contacts	4.76	8	0.78
Perceived quality of social support	6.54	8	0.59
Confidence in health care services	4.98	8	0.76
*Sixth COVID-19 Health Survey*			
Satisfied with social contacts	13.31	8	0.10
Perceived quality of social support	12.78	8	0.12
Social loneliness	6.97	8	0.55
Emotional loneliness	12.68	8	0.12

**Table 6:** Association between domestic violence and subjective social isolation by means of crude and adjusted^&^ OR (95% CI), stratified by the second and sixth COVID-19 Health Survey, Belgium 2020–2021

Domestic violence	Second survey (April 2020)		Sixth survey (March 2021)	
	Crude OR (95% CI)	Adjusted OR (95% CI)	Crude OR (95% CI)	Adjusted OR (95% CI)
Satisfied with social contacts				
(Rather) unsatisfied	1.43 (1.24–1.66)^***^	1.25 (1.08–1.44)^*^	1.51 (1.26–2.80)^***^	1.30 (1.08–1.56)^*^
(Rather) satisfied	1	1	1	1
Perceived quality of social support				
Weak support	2.52 (2.20–2.87)^***^	2.26 (1.97–2.58)^***^	2.79 (2.37–3.27)^***^	2.41 (2.04–2.84)^***^
Moderate/strong support	1	1	1	1
Confidence in health care services				
Very confident	1	1	–	–
Less confident	1.59 (1.30–1.96)^***^	1.38 (1.13–1.71)^*^	–	–
Social loneliness				
Not lonely	–	–	1	1
Lonely	–	–	2.98 (2.96–3.00)^***^	2.64 (2.23–3.13)^***^
Emotional loneliness				
Not lonely	–	–	1	1
Lonely	–	–	2.64 (2.16–3.24)^***^	2.22 (1.80–2.73)^***^

^&^: controlled for the covariates

*: *p* < .05

**: *p* < .001

***: *p* < .0001

## Discussion

Compared to the HIS 2018 findings, domestic violence increased in frequency in Belgium since the beginning of the coronavirus crisis and the persistent confinement measures even worsened this situation. In April 2020, 6 weeks after the introduction of the confinement measures, adults were 3 times more likely to report being victim of domestic violence than in 2018, and in March 2021, they were even 5 times more likely to report it than in 2018. However, the longer reference period for reporting domestic violence between the two COVID-19 Health Surveys (retrospectively in the past 6 weeks versus in the past year) may explain the increased risk of being a victim of domestic violence from 6 weeks to 1 year after the coronavirus crisis. As is the case in other studies [7, 38, 39], psychological violence was the most frequent form of domestic violence reported in our study. This form of violence can definitely also be experienced as traumatic, but is often regarded by society as less serious than physical violence [2]. However, psychological violence can go hand in hand with physical violence [9, 14]. In this study, most of the victims reporting physical violence (including sexual violence) also experienced psychological violence. So even though confinement is in the interest of curbing the virus transmission, it can put people in a potentially dangerous situation or even put victims more at risk [8, 14, 15, 18, 19, 32].

The association between domestic violence and social isolation was assessed based on five subjective social isolation indicators. The main conclusion is that there is an association between being a victim of domestic violence and perceived social isolation. Victims of domestic violence were twice as likely to indicate having weak social support in the first 6 weeks of confinement, as well as 1 year after the introduction of the confinement measures. This is in line with a study that showed that battered women perceived their social support as being weak [21]. Besides, 6 weeks and one year after the confinement, victims of domestic violence were also more likely to indicated being unsatisfied by their social contacts. Furthermore, in the beginning of the confinement, victims of domestic violence were more likely to have less confidence in health care services. In general, in the beginning of the confinement period there was a reduced accessibility to health care and social services and later there was a switchover to virtual consultations [1, 8]. In normal circumstances, victims are already reluctant to seek help, so confinement could make it even more difficult for them [20]. These virtual technologies can of course be useful; however, the victim cannot always use them discreetly or cannot access them because of control tactics by the perpetrator. On the other hand it may also be possible that acquiring such technologies is too expensive [14]. Other explanations of this association between being victim of domestic violence and experiencing more social isolation is that perpetrators often disrupt the friendship ties of the victim [21] or that they use COVID-19 as a coercive control mechanism to make their victim anxious which will make them stay at home [17]. Nevertheless, even though the confinement measures have been loosened over time, 1 year after their introduction victims of domestic violence are twice as likely to feel lonely, both socially and emotionally, than nonvictims.

Not only does the taboo on domestic violence make it difficult for victims to report it or to seek help [9, 16], the confinement clearly creates other barriers (i.e. movement restrictions, trapped with perpetrator, reduced access to social services) as well [15]. Consequently there are vulnerable people who do not get the help they need which will affect their safety, health and wellbeing [14, 38]. When confinement is necessary, policy makers should take measures that are as bearable as possible for everyone [40]. Therefore it is essential to provide adequate support during confinement [8, 20]. In this context the United Nations evoked to prioritise support and warning systems for victims of domestic violence [17]. Support can come from different areas. For example, health care and social professionals, often the first point of contact for victims, must be made aware of the increased risk of domestic violence during confinement. Training can help them recognize this problem (signs and people at risk) and anticipate in an appropriate way to guarantee the safety of the victim [1, 16, 19]. Governments should also invest in mental health care (expand and free of charge), as victims of domestic violence often have to deal with psychological problems [7, 19, 32]. It is also crucial that social support systems and shelters remain accessible during confinement [12, 16]. In Belgium, hotels and empty government buildings were offered to victims as an alternative for shelters [18]. Of course the general population needs to be informed about the available services like hotlines, online platforms and shelters through (social) media and health and social services [16, 17]. Regarding online platforms, it is important that they contain a mechanism to quickly and safely exit them so that these platforms cannot be tracked by the perpetrator [16]. Still, it is important that people keep connected with family, friends and neighbors during confinement which can help to reduce social isolation among victims of domestic violence and facilitate a faster report of a concern of domestic violence [16–18]. In Belgium, but also in some other European countries, the government has set up a warning system in pharmacies. The purpose of this system is that victims can alert the staff that they are in danger and need support by using a code word [12, 17–19]. In addition, it is also important to focus on prevention of domestic violence by, among others, promoting gender equality [8, 9, 12, 18].

The first strength of this study is that it was conducted in the heat of the battle against the coronavirus, this goes especially for the second COVID-19 Health Survey (organised 6 weeks after the introduction of the confinement measures). This rapid approach could only be done by using a web survey, which was accessible by mobile phone, a tablet and computer [25]. An advantage of a web survey is that it is very user-friendly and the high quality data are readily available [41, 42]. Another strength of these online health surveys is the repeated cross-sectional way of data collection and the fact that sensitive topics such as domestic violence can be surveyed [2]. The last strength is that domestic violence was investigated in a large convenience sample at national level among the population of 18 years and older.

However, online surveys also introduce weaknesses in the study design. For instance children, who can also be a victim of domestic violence, were not included in this study. This was a restriction imposed by the Ethics Committee. Other weaknesses of this study are the selection bias (e.g. the digital divide: overrepresentation of higher educated people and internet literate people), the potential healthy volunteer bias, (e.g. need for good concentration level to complete questionnaire) and misreporting since self-reported data are subject to social desirability [2, 25]. Besides, the reliability of the questions on domestic violence can be affected because they were developed without pretesting due to the urgency [25]. Finally, it also needs to be noted that domestic violence in the HIS 2018 was questioned differently than in the COVID-19 Health Surveys (with different time periods): in the former, victims of violence were asked who the perpetrator was (stranger(s), colleague(s), acquaintance(s), friend(s), (ex-) partner, parent(s), (plus) child (ren) or family member). In addition, the methodology was also different: a survey on household level where maximum 4 members were questioned by an interviewer, next to a paper questionnaire (including the more sensitive items like violence) which needed to be filled in by the members themselves [26].

## Conclusion

In Belgium, the prevalence of domestic violence increased during the period of confinement imposed to slow the coronavirus circulation. Moreover, an association between being a victim of domestic violence and experiencing more social isolation was determined. Although confinement is needed to counteract the virus transmission, it increases the barriers to report domestic violence and to seek help. Consequently, it can put people in a dangerous situation since they do not get the help they need. Policy makers should provide adequate support and warning systems during confinement. Finally, the population must be made aware that domestic violence is not tolerated in society.

## Data Availability

Access to the data of the second and the sixth Belgian COVID-19 health survey reported in this manuscript can be requested by sending an e-mail to HIS@sciensano.be.

## Abbreviations

HIS: Health Interview Survey
U.S.: United States
GDPR: General Data Protection Regulation
OR: Odds Ratio
CI: Confidence interval
